# Rare Metastatic Involvement of the Sigmoid Mesocolon by Low‐Grade Endometrial Stromal Sarcoma

**DOI:** 10.1155/crog/4460648

**Published:** 2026-09-17

**Authors:** Niloufar Ahmadloo, Susan Andalibi, Ahmad Mosalaei, Shapour Omidvari, Mansour Ansari, Heshmatollah Salahi, Nazanin Karamifar, Ehsan Mohammad Hosseini

**Affiliations:** ^1^ Department of Radiation Oncology, Namazi Hospital, Shiraz University of Medical Sciences, Shiraz, Fars Province, Iran, sums.ac.ir; ^2^ Department of Surgery, Namazi Hospital, Shiraz University of Medical Sciences, Shiraz, Fars Province, Iran, sums.ac.ir; ^3^ Department of Pathology, Shiraz University of Medical Sciences, Shiraz, Fars Province, Iran, sums.ac.ir; ^4^ Department of Neurosurgery, Namazi Hospital, Shiraz University of Medical Sciences, Shiraz, Fars Province, Iran, sums.ac.ir

**Keywords:** colon metastasis, endometrial stromal sarcoma, hormonal therapy, low-grade ESS, mesocolon

## Abstract

**Introduction:**

Endometrial stromal sarcoma (ESS) is an uncommon uterine malignancy with an indolent course and potential for late recurrence or metastasis. Extrauterine ESS is exceedingly rare, especially in the absence of a primary uterine lesion.

**Case Presentation:**

We present a case of a 50‐year‐old woman with low‐grade ESS metastasizing to the sigmoid mesocolon. The patient underwent surgical resection followed by radical hysterectomy, bilateral salpingo‐oophorectomy, and hormonal therapy with letrozole. After 5 years of follow‐up, she remains disease‐free.

**Discussion:**

This case underscores the importance of considering ESS in the differential diagnosis of mesocolon masses and highlights the role of combined surgical and hormonal therapy in achieving long‐term remission.

**Highlights**

• Low‐grade endometrial stromal sarcoma (LG‐ESS) is a rare uterine malignancy with potential for late metastasis.
• Metastatic LG‐ESS to the colon or mesocolon is exceptionally uncommon.
• Accurate diagnosis requires a combination of histopathology and immunohistochemistry.
• Hormonal therapy, including aromatase inhibitors, can be effective in managing recurrent or metastatic disease.
• Lifelong surveillance is recommended because of the tumor′s high recurrence potential.

## 1. Introduction

Endometrial stromal sarcoma (ESS) is an uncommon neoplasm, representing approximately 0.2%–0.5% of all uterine malignancies and 10%–15% of primary uterine sarcomas [1]. Development of ESS within endometriotic lesions has been documented only rarely. Extrauterine ESS may arise in the ovary, fallopian tube, pelvic cavity, colon, or retroperitoneum. Because of its rarity, the pathogenesis of ESS remains incompletely understood. The tumor typically exhibits an indolent clinical course with a propensity for local recurrence. Although colorectal carcinoma is the most frequent malignancy of the gastrointestinal tract, secondary metastatic tumors involving the colon are rare, comprising approximately 1% of all colorectal cancers [2].

Herein, we report a rare case of low‐grade ESS (LG‐ESS) metastasizing to the sigmoid mesocolon.

## 2. Case Presentation

**Figure 1:**
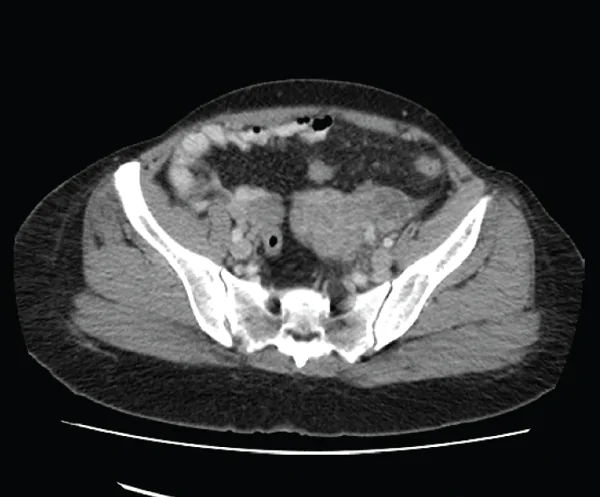
43 × 40 mm heterogeneously enhancing soft‐tissue density in the left mesocolon with fat stranding.

A 50‐year‐old multiparous woman (G5P5A0) with no notable past medical or family history initially presented with severe abdominal pain 6 months before the eventual diagnosis. She improved with oral analgesics and was discharged without additional intervention. Six months later, she returned with acute, persistent left lower quadrant abdominal pain refractory to parenteral analgesics. Physical examination revealed abdominal pain and lower abdominal tenderness without rebound tenderness. Laboratory evaluation, including complete blood count and serum biochemistry, was unremarkable. Transabdominal and pelvic sonography demonstrated a normal uterus and left ovary with an endometrial thickness of approximately 3 mm. A hypoechoic cystic lesion was noted in the left ovary, along with thickening of a bowel loop wall and marked surrounding fat stranding in the left lower quadrant, initially interpreted as possible diverticulitis. For further evaluation, spiral chest, abdominal, and pelvic computed tomography (CT) was performed. Chest CT was unremarkable, whereas abdominopelvic CT revealed a 43 × 40 mm heterogeneously enhancing soft‐tissue mass in the left mesocolon with associated fat stranding, raising suspicion for malignancy (Figure 1).

**Figure 2:**
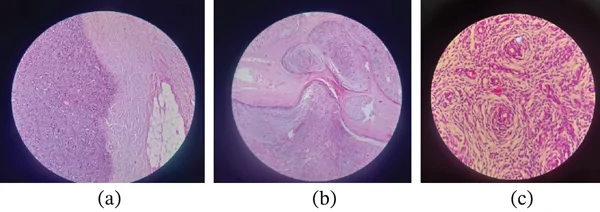
(a) Low‐power appearance of endometrial stromal sarcoma metastatic to the large bowel wall. (b) Extensive muscular permeation by sharply defined tumor islands with pointed edges. (c) Typical microscopic appearance of endometrial stromal tumor showing band‐oval cells arranged concentrically around a spiral arteriole.

Under a presumptive diagnosis of intestinal malignancy, the patient underwent laparotomy including low anterior resection; mobilization of the sigmoid colon, upper rectum, splenic flexure, and transverse colon; excision of regional lymph nodes and partial omentum; and rectosigmoid anastomosis. Pathological examination identified a 5 × 3.5 × 2.5 cm tumor located within the sigmoid mesocolon, consistent with metastatic LG‐ESS. Surgical margins were free, and no involvement of the mucosa, submucosa, muscularis, or regional lymph nodes was noted, which indicates stage IVA. Immunohistochemistry confirmed the diagnosis (CD10‐positive, SMA‐ and inhibin‐negative, and Ki‐67 1%–2%) (Figure 2).

**Figure 3:**
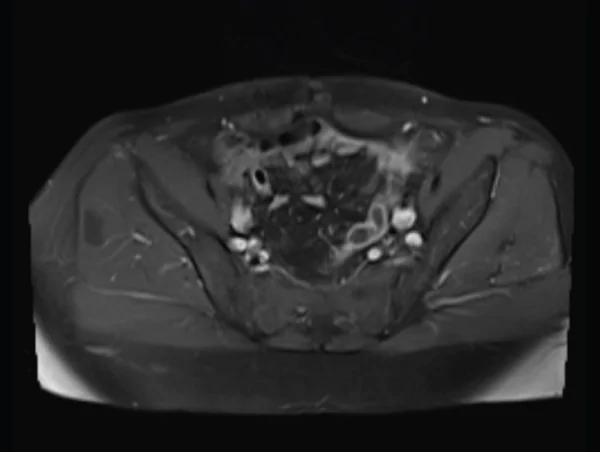
Posttreatment MRI with gadolinium, performed 5 years after treatment, showed no signs of recurrence.

Subsequently, the patient underwent laparoscopic radical hysterectomy with bilateral salpingo‐oophorectomy and pelvic lymphadenectomy. Histology revealed chronic cervicitis and leiomyoma of the myometrium without other abnormal findings. She received adjuvant hormonal therapy with the aromatase inhibitor letrozole 2.5 mg daily for 2 years. At 5‐year follow‐up, abdominal and pelvic MRI demonstrated no evidence of recurrence or metastasis (Figure 3).

## 3. Discussion

ESS is an exceedingly rare uterine malignancy, accounting for approximately 0.2% of all uterine tumors and 10%–15% of primary uterine sarcomas [3], with an estimated annual incidence of one to two cases per million women. It typically affects younger women (average age 42–58 years), with 10%–25% occurring in premenopausal individuals [2]. Primary extrauterine ESS is extremely uncommon in the absence of direct extension or metastasis from a uterine primary. The etiology of ESS is largely unknown. Key differential diagnoses include ovarian sex‐cord stromal tumors with a predominant spindle cell component, Sertoli–Leydig cell tumors, and primary mesenchymal tumors such as gastrointestinal stromal tumors (GISTs) [4–6]. Immunohistochemical profiling with CD10, desmin, ER, and PR helps distinguish these entities [7].

In 2014, the World Health Organization (WHO) classified endometrial stromal tumors into four categories: endometrial stromal nodule (ESN), LG‐ESS, high‐grade ESS (HG‐ESS), and undifferentiated uterine sarcoma (UUS) [8]. ESS represents a genetically heterogeneous group of tumors with recurrent chromosomal translocations. The JAZF1–SUZ12 translocation is characteristic of LG‐ESS and absent in other uterine mesenchymal neoplasms [9]. LG‐ESS is a slow‐growing, hormone‐sensitive malignancy with an indolent nature, yet local recurrences and distant metastases can occur even 20 years after the initial diagnosis [2], affecting approximately 30% of patients. Common sites include the vagina, pelvis, and peritoneal cavity [10], with less frequent involvement of the lungs, liver, bladder, breast, heart, brain, and bones [3, 11]. Metastatic spread to the bowel usually occurs via peritoneal seeding, though hematogenous and lymphatic dissemination have also been reported [2].

Management of LG‐ESS requires a multidisciplinary approach. Surgery remains the cornerstone, typically a total abdominal hysterectomy with bilateral salpingo‐oophorectomy. Lymph node dissection provides prognostic information but does not improve survival, so its need is uncertain. Systemic lymph node dissection may not be recommended in patients with ESS unless the patient has extraurinary involvement and a clinically suspicious enlarged node or advanced sarcomas, and there is no consensus regarding omentectomy in surgery performed for endometrial cancer. Adjuvant therapies—radiotherapy, chemotherapy, hormone therapy, or observation—are individualized [12–14]. Because LG‐ESS expresses estrogen and progesterone receptors, hormonal agents such as progestins, third‐generation aromatase inhibitors, and gonadotropin‐releasing hormone analogs are effective [2]. Progestin therapy is considered first‐line for residual or recurrent disease, with stabilization or regression reported in more than 50% of treated patients [14]. Reported 5‐year survival rates for LG‐ESS range from 67% to 100%. Recurrent and metastatic disease may be successfully treated with surgical excision, radiation, progestin therapy, or combination regimens [15]. Owing to the high potential for late recurrence, lifelong surveillance is recommended.

## 4. Conclusion

ESS is a rare uterine tumor, and metastatic involvement of the colon is an exceptionally uncommon presentation. Because of the low incidence of this entity, optimal management remains uncertain and requires an expert multidisciplinary team. Nevertheless, recurrent and metastatic LG‐ESS can often be effectively managed with surgical excision, radiation, progestin therapy, or multimodal approaches. Our case illustrates a rare instance of LG‐ESS metastasizing to the rectum, highlighting the importance of thorough evaluation and long‐term follow‐up.

## Funding

No funding was received for this manuscript.

## Disclosure

All authors have read and approved the final version of the manuscript. Susan Andalibi, the corresponding author, had full access to all of the data in this study and takes complete responsibility for the integrity of the data and the accuracy of the data analysis.

## Consent

Written informed consent was obtained from the patient for publication of this case report and any accompanying images.

## Conflicts of Interest

The authors declare no conflicts of interest.

## Data Availability

The data that support the findings of this study are available on request from the corresponding author. The data are not publicly available due to privacy or ethical reasons.
